# Identification of EMT-related high-risk stage II colorectal cancer and characterisation of metastasis-related genes

**DOI:** 10.1038/s41416-020-0902-y

**Published:** 2020-05-21

**Authors:** Kai Wang, Kai Song, Zhigang Ma, Yang Yao, Chao Liu, Jing Yang, Huiting Xiao, Jiashuai Zhang, Yanqiao Zhang, Wenyuan Zhao

**Affiliations:** 1 Department of Systems Biology, College of Bioinformatics Science and Technology, Harbin Medical University, Harbin, 150086 China; 2 Department of Gastrointestinal Medical Oncology, Harbin Medical University Cancer Hospital, No. 150, Haping Road, Nangang District, Harbin, 150001 China

**Keywords:** Tumour biomarkers, Microarray analysis

## Abstract

**Background:**

Our laboratory previously reported an individual-level prognostic signature for patients with stage II colorectal cancer (CRC). However, this signature was not applicable for RNA-sequencing datasets. In this study, we constructed a robust epithelial-to-mesenchymal transition (EMT)- related gene pair prognostic signature.

**Methods:**

Based on EMT-related genes, metastasis-associated gene pairs were identified between metastatic and non-metastatic samples. Then, we selected prognosis-associated gene pairs, which were significantly correlated with disease-free survival of stage II CRC using multivariate Cox regression model, as the EMT-related prognosis signature.

**Results:**

An EMT-related signature composed of fifty-one gene pairs (51-GPS) for prediction-relapse risk of patients with stage II CRC was developed, whose prognostic efficiency was validated in independent datasets. Moreover, 51-GPS achieved better predictive performance than other reported signatures, including a commercial signature Oncotype Dx colon cancer and an immune-related gene pair signature. Besides, EMT-related functional gene sets achieved high enrichment scores in high-risk samples. Especially, loss-of-function antisense approach showed that DEGs between the predicted two clusters were metastasis-related.

**Conclusions:**

The EMT-related gene pair signature can identify the high relapse-risk patients with stage II CRC, which can facilitate individualised management of patients.

## Background

Colorectal cancer (CRC) ranks third in terms of incidence, but second in terms of cancer-related death worldwide.^1^ Treatment decision and prognosis assessment mainly depend on the pathological stage of the tumour.^2^ However, about 20% patients of stage II CRC will relapse after curative surgery.^3^ Therefore, some other factors were proposed for therapy decisions. For example, stage II patients with high-risk factors, such as T4 stage and high tumour grade, have a greater chance of relapse and should be treated with chemotherapy after surgery.^4^ But these clinicopathological risk factors do not adequately distinguish between patients who have high or low risk of relapse, and lead to over- or under-diagnosis.^5^

Several studies have developed quantitative signatures based on gene expression for survival stratification with stage II CRC,^6,7^ which were developed from genome-wide, prognosis-related and immune-related genes. Unfortunately, the clinical practice is limited owing to issues such as overfitting on small discovery datasets and lack of sufficient validation. Besides, this type of prognostic signature calculated by the sum of the weighted expression values of the characteristic genes is difficult to be reproducible due to experimental batch effects and platform differences.^8,9^ In addition, gene expression measurements are greatly affected by the sampling locations^10,11^ and RNA degradation problem during sample preparation^12^ of tumour tissues. Although a quantitative signature Oncotype Dx colon cancer has been used commercially, some patients are categorised as “intermediated risk” cluster, which complicates clinical decision-making. To tackle the above-mentioned problems, qualitative methods, such as TSP^13^ and k-TSP,^14^ have been proposed, which are relatively robust to these factors. Using this method, several qualitative signatures have been developed for prognosis/prediction of tumours. Especially for CRC patients, based on the within-sample relative expression orderings (REOs) of genes, our laboratory previously reported an individual-level prognostic signature consisting of three gene pairs for predicting the post-surgery relapse risk of stage II CRC.^15^ This signature was developed by training on microarray expression data, and validated using independent microarray datasets. However, this signature was not assessed in the RNA-seq platform. Wu et al.^16^ have also constructed REO-based individualised prognostic signatures. Nevertheless, the signature was developed without considering the specificity of stage.

The epithelial-to-mesenchymal transition (EMT) is a centrally important mechanism for the metastasis of carcinomas,^17^ which was typically characterised by loss of cell–cell adhesion and apical-based cell polarity, as well as the increased invasion of cells.^18^ Furthermore, induction of EMT has been reported to lead to patients at an early-stage CRC more prone to metastasis.^19,20^ Herein, we aim to construct a gene pair signature to figure outpatients at risk of relapse using EMT-related genes in stage II CRC.

## Methods

### Data acquisition and pre-processing

Twelve CRC gene expression datasets were collected from the public database, including ten microarray datasets and a RNA-seq dataset from the Gene Expression Omnibus (GEO, https://www.ncbi.nlm.nih.gov/geo/),^21^ and one RNA-seq dataset from The Cancer Genome Atlas (TCGA, https://www.cancer.gov/about-nci/organization/ccg/research/structural-genomics/tcga),^22^ as described briefly in Supplementary Table S1a. Specific clinicopathological features are described in Supplementary Table S1b. For the datasets from GEO, we downloaded the raw data (.CEL files) and used the robust multi-array average (RMA) method^23^ for background adjustment without quantile normalisation. Each probe ID was mapped to Entrez gene ID with the corresponding platform files. If a probe was mapped to multiple or zero genes, the data of this probe were discarded. If multiple probes were mapped to the same gene, the expression level of this gene was summarised as the arithmetic mean of the values of multiple probes. For TCGA transcriptional data derived from Illumina high-throughput sequencing (HiSeq) platform, the raw count and fragments per kilobase of transcript per million fragments mapped (FPKM) values were extracted. For mutation data derived from the Illumina Genome Analyzer DNA Sequencing GAIIx platform, only the nonsynonymous mutations remained. Data of copy number variations (CNVs) were processed with the GISTIC algorithm.^24^ Samples from GSE39582 and TCGA were used as training cohorts due to their relatively high-quality clinical records and long-term follow-up. Datasets with sample size more than 50 were used as independent validation cohorts. However, as for small-sample datasets with sample size less than 50, we combined them according to the individual platforms, and defined Com_570 and Com_96, since different datasets could be directly integrated based on the within-sample REOs.^13^

### Construction of an individualised prognostic signature based on EMT-related genes

**Fig. 1:**
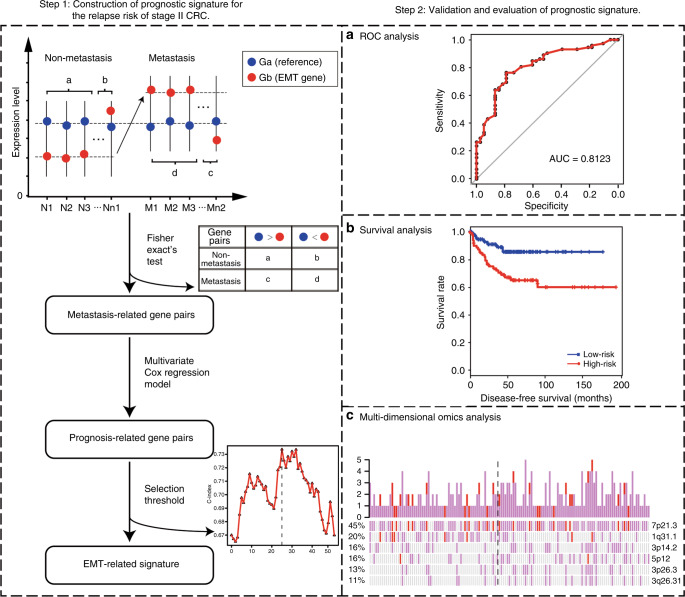
Flowchart of the processes for developing and validating the prognosis signature. The first step is to construct a prognostic signature for the relapse risk of stage II CRC. The second step is to verify the prognostic signature through ROC analysis, survival analysis and multi-dimensional omics analysis.

Figure 1 describes the processes for developing and validating the prognostic signature. Firstly, we collected EMT-related genes from public databases (dbEMT: http://dbemt.bioinfo-minzhao.org/,^25^ MSigDB: http://software.broadinstitute.org/gsea/msigdb/index.jsp) and literature (Liang et al.^26^). Secondly, we identified stable expressed genes across metastatic and non-metastatic samples with coefficient of variation (CV) less than 0.3,^27^ which were defined as reference genes. In order to calculate the CV, the FPKM values from TCGA and probe intensities from microarray were log_2_ transformed,^28^ and FPKM values less than 1 were set to 1.

Thirdly, among gene pairs composed of EMT-related and reference genes, two genes in a gene pair, *a* and *b*, with expression values of *G*_*a*_ and *G*_*b*_, the Fisher’s exact test was used to identify metastasis-associated gene pairs whose frequency of samples with the REO pattern *G*_*a*_ < *G*_*b*_ (or *G*_*a*_ > *G*_*b*_) was significantly higher in the metastatic CRC than the non-metastatic CRC samples. Then, for each of the metastasis-associated gene pairs, we used the multivariate Cox regression model to identify prognosis-associated gene pairs whose REOs were significantly correlated with disease-free survival (DFS) of stage II CRC samples treated only with curative surgery in GSE395892, which were supposed as the candidate relapse-risk signature. Further, we calculated the concordance index (C index)^29^ of each possible threshold from one to the number of gene pairs, and selected the one (*k*) that could reach the largest C index in the training data as the appropriate threshold of the signature. A sample was classified as a high-risk cluster if at least *k* gene pairs voted for high-risk, otherwise, low-risk cluster. The prognosis-associated gene pairs with the appropriate threshold were defined as the gene pair signature (GPS), which could be used directly in the validation datasets.

### Survival analysis

DFS was defined as the time from surgery to relapse or the final documented data (censored). Survival curves of DFS between different clusters were estimated using the Kaplan–Meier (K–M) method, the differences between the survival curves were compared using the log-rank test^30^ and 95% confidence intervals (CIs) were calculated using a univariate Cox regression model.^29^ The independent prognostic value of the signature was assessed by multivariate Cox regression model after adjustment for clinical factors. The predictive accuracy of the signature was assessed using the receiver-operating characteristic curve (ROC, “pROC” package, version 1.14.0). All statistical analyses were performed using R software version 3.5.2 (https://www.r-project.org/).

### Functional enrichment analysis

We performed gene set enrichment analysis using GSEA software (http://software.broadinstitute.org/gsea/index.jsp) with 1000 permutations. For the RNA-seq data from TCGA, FPKM values were used to calculate the log_2_ fold change between high- and low-risk clusters. The hallmark gene sets were used for the target gene sets for GSEA. The gene sets satisfying *p* < 0.05 were considered statistically significant. Besides, we assessed consensus molecular subtype (CMS) classification between high- and low-risk clusters using the “CMSclassifier” package, version 1.0.0.

### Cell lines and transfection

HCT116 cells were purchased from the American Type Culture Collection (Manassas, VA, USA) and cultured at 37 °C in a humified atmosphere of 95% O_2_ and 5% CO_2_. HCT116 cells were grown in high-glucose DMEM medium (Thermo Fisher Scientific, Waltham, MA, USA) with 10% foetal calf serum (Thermo Fisher Scientific, Waltham, MA, USA). ShRNA plasmids were purchased from Vigene Biosciences. Transfections (0.5 µg of shRNA plasmid) were performed using the Lipofectamine^®^ 2000 kit (Thermo Fisher Scientific, Inc.) according to the manufacturer’s protocol.

### Western blotting and wound-healing assay

Total proteins were harvested from cultured cells using an ice-cold lysis buffer. Proteins were separated by 10% SDS/PAGE and then transferred to PVDF membranes. The membranes were blocked with 5% non-fat milk, and then incubated with primary antibodies and β-actin (Proteintech, Chicago, USA), followed by horseradish peroxidase (HRP)-conjugated secondary antibodies (Proteintech). Immunoreactive proteins were detected using a chemiluminescence solution (Thermo Fisher Scientific).

HCT116 cells were transfected for 24 h and seeded in six-well plates and incubated until they were 90% confluent. A straight scratch was then made across the base of the well. Images of the cells were captured at ×40 magnification (Nikon, Tokyo, Japan) at 0 and 24 h, and used to determine cell migration. The width of the wound was measured by ImageJ, and the data were used to quantify the rate of cell migration. Each experiment was independently performed in triplicate.

### Genomic data analysis

Fisher’s exact test was used to detect genes that had significantly different mutation or CNV frequencies. Significantly differentially expressed genes were identified between high- and low-risk clusters by edgeR algorithm. OncoPrint^31^ was used to show top 50 nonsynonymous mutant genes and significant CNVs between the two risk clusters.

## Results

### Construction of prognostic signature for the relapse risk of stage II CRC

Firstly, we validated prognostic signature previously constructed by our laboratory in the RNA-seq dataset from TCGA. Unfortunately, this signature (3-GPS) was not applicable (Supplementary Table S2). Then, we constructed an EMT-related prognostic signature for predicting post-surgery relapse risk of stage II CRC.

**Fig. 2:**
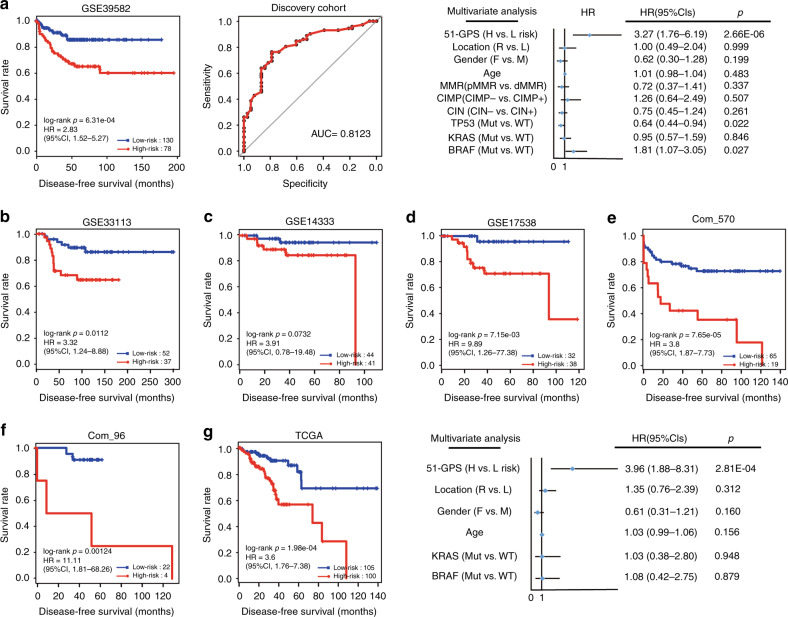
The performance of the 51-GPS in discovery and validation cohorts. **a** The Kaplan–Meier (K–M) curve of DFS for patients between high- and low-risk clusters in the discovery cohort (left); *p* values comparing risk clusters were calculated with the log-rank test. The assessment of the predictive consistency of signature via the AUC curve (middle). Multivariate Cox regression model was performed to assess the prognostic efficiency of 51-GPS (right). Solid dots represent the HR, and the open-ended horizontal lines represent the 95% CIs. **b**–**f** The validation of the prognostic capacities of 51-GPS in independent microarray datasets. **g** The K–M curve of DFS and multivariate Cox regression analysis in RNA-seq data.

We collected a list of 1250 EMT-related genes from public databases and literature, which involved 782 genes on the platform of the discovery cohort. Then, we extracted 6610 stable expressed genes (CV < 0.3) across metastatic samples (stage III and IV CRC) and non-metastatic samples (stage I CRC) simultaneously in GSE39582 and TCGA, which were defined as reference genes. Among gene pairs composed of EMT-related and reference genes, we identified 31,603 and 44,814 metastasis-associated gene pairs between metastatic and non-metastatic samples in the two datasets (Fisher’s exact test, adjusted *p* < 0.05), respectively. The two lists of gene pairs had 1726 overlaps, and 99.9% (1725) of them had the same reversal patterns. Among the 1725 gene pairs, we identified 51 prognosis-associated gene pairs correlated with DFS of 208 stage II CRC samples treated with surgery only in the GSE39582 dataset after adjusting for clinical factors, including location and KRAS status (multivariate Cox regression model, *p* < 0.05). The 51 prognosis-associated gene pairs were defined 51-GPS (Supplementary Table S3a). For all possible thresholds from 1 to 51, the largest C index was 0.734 when threshold was 25. A sample was classified as a high-risk cluster if at least 25 gene pairs voted for the high-risk, otherwise, low-risk cluster. Using this signature, 120 stage II CRC patients in the discovery cohort of GSE39582 were predicted to be at low post-surgery relapse risk, which had a significantly better DFS than the 78 patients who were predicted to be at high post-surgery relapse risk (Fig. 2a, HR = 2.83, 95% CI: 1.52–5.27, log-rank *p* = 6.31E−04). For dividing stage I, III and IV samples of GSE39582, the AUC was 0.812 compared with the original labels of the metastatic and non-metastatic samples (Fig. 2a). Besides, the 51-GPS remained a powerful prognostic factor after adjustment for the clinical factors (multivariate Cox regression model, *p* < 0.05, Fig. 2a).

### Validation and evaluation of the 51-GPS

Then, we applied the signature to independent datasets to validate the prognostic value. In the validation cohort with 89 stage II CRC patients treated with surgery only from the GSE33113, 52 patients were predicted to be in the low-risk cluster, whose DFS was significantly higher than the other 37 patients who were predicted to be in the high-risk cluster (Fig. 2b, HR = 3.32, CI: 1.24–8.88, log-rank *p* = 0.0112). A similar result was shown in GSE14333 and GSE17538 cohorts (Fig. 2c, d). Since different datasets could be directly integrated based on the within-sample REOs, we combined datasets with small samples (sample size < 50) according to the individual platforms and defined Com_570 and Com_96, respectively. As expected, patients in the Com_570, combined from the GSE26906, GSE31595, GSE39084 and GSE92921 cohorts, were significantly stratified in terms of DFS (Fig. 2e). However, only 39 gene pairs (Supplementary Table S3b) of 51-GPS were detected by Com_96 from the HG-U133A Array, we recalculated the optimal vote threshold in the training dataset as described above and a sample was classified as a high-risk cluster if at least 20 gene pairs voted for the high-risk, otherwise, low-risk cluster. Patients in the Com_96, combined from the GSE12945 and GSE41258 cohorts, were also stratified into two risk clusters with significant DFS differences by the signature (Fig. 2f).

While for 205 samples of stage II CRC patients measured by RNA-seq in TCGA, 105 patients were predicted to be at low post-surgery relapse risk, which had a significantly better DFS than the 100 patients who were predicted to be at high post-surgery relapse risk (HR = 3.6, 95% CI: 1.76–7.38, log-rank *p* = 1.98E−04). Multivariate Cox analyses demonstrated that 51-GPS was an independent predictive factor after adjusting for the clinical factors (Fig. 2g). In the above independent microarray datasets, 51-GPS performed comparably with the prognostic signature previously constructed by our laboratory (Supplementary Table S2).^15^ Even for the RNA-seq dataset where the previous signature was not applicable, 51-GPS still showed distinct prognostic differences. We also used 51-GPS to predict the risk cluster of the GSE50760 dataset, which consisted of 54 samples (normal colon, primary CRC and liver metastasis) generated from 18 CRC patients (Table 1). There were 23 of 36 tumour samples predicted as high-risk cluster, with 15 liver metastatic samples predicted as high-risk cluster, and all of 18 normal samples were predicted as low-risk cluster. Comprehensively, the EMT-related signature (51-GPS) was a valuable prognostic factor with robust predictive power.

**Table 1:** The predicted risk cluster of samples in GSE50760.

Pathology	Primary CRC	Liver metastasis	Normal colon
predicted			
High risk	8	15	0
Low risk	10	3	18

### Comparison with other known prognostic signatures

**Fig. 3:**
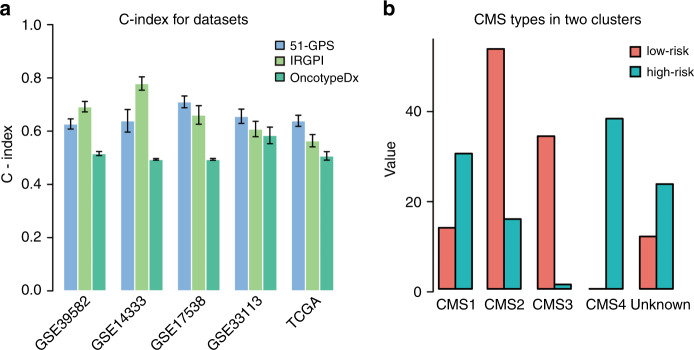
Comparison of 51-GPS with other transcriptome-based signatures. **a** The comparison of predictive performance for 51-GPS, PRGPI and Oncotype Dx colon cancer. The error bars above the bar graph represent the standard deviation of the C index. **b** The CMS classification between high- and low risk. The vertical coordinate represents the number of CMS subtypes.

To further explore the predictive efficiency of the newly developed signature, we compared the 51-GPS with other reported signatures, including Oncotype Dx colon cancer and an immune-related gene pair signature (IRGPI). Oncotype Dx colon cancer, which is a quantitative transcriptional signature consisting of 12 genes, has been used commercially for predicting post-surgery relapse risk of stage II and III CRC. The IRGPI is also a qualitative transcriptional signature based on immune-related genes. As for microarray datasets, 51-GPS and IRGPS got comparative results for the survival differences, which all achieved a higher C index than Oncotype Dx for both training and validation datasets (Fig. 3a). Of note, for the RNA-seq dataset from TCGA, only 51-GPS divided patients into two risk clusters with a significant survival difference, and obtained the largest C index among three signatures. In summary, 51-GPS was a robust qualitative transcription prognostic signature with a better predictive efficiency than other known signatures.

### EMT-related functional gene sets enriched in the high-risk cluster

Stage II CRC patients of the TCGA cohort were divided into different relapse-risk clusters according to the 51-GPS. We performed gene set enrichment analysis (GSEA) between these two risk clusters. Twenty-two gene sets were significantly enriched in the high-risk cluster with *p* values less than 5% (Supplementary Table S4). Among 22 gene sets, the epithelial–mesenchymal transition gene set had the highest enrichment score (ES) among all gene sets. Other known gene sets associated with EMT were also significantly enriched in the high-risk cluster, including “Apical junction”^17^, “KRAS signalling”^32^ and “TGF beta signalling”^33^, which play important roles in poor outcome of CRC patients. For example, cell polarity is defined by apical cell–cell tight junctions, which can induce EMT directly, and provide tumour cells with the ability to escape from the primary tumour to distant regions.^17^ TGF beta is a pleiotropic cytokine that regulates cell proliferation, apoptosis, differentiation, migration and invasion, which has also been reported to play a crucial role in EMT.^33^ Besides, assessing CMS classification between high- and low risk (Fig. 3b), we found that the number of CMS4 subtype associated with EMT, was significantly less in the low- than in the high-risk cluster (Fisher’s exact test, *p* = 2.39E−11).

### Validation of the functions of metastasis-related genes

**Fig. 4:**
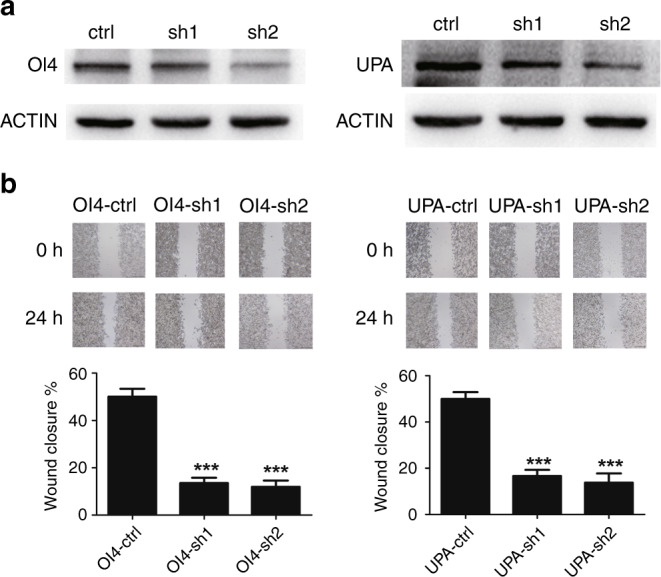
ShRNA lentivirus suppressed CRC cell proliferation and metastasis in vitro. Representative images of western blotting (**a**) and cell wound-healing assays (**b**) of OI4 and UPA after transfection with shRNA lentivirus compared with controls.

To further explore the mechanism of tumour metastasis and identify possible drug targets, differentially expressed genes were identified between high- and low-risk samples in stage II CRC of TCGA. We found that most of them have been reported to be associated with CRC metastasis, such as “S100A2”^34^, “ANXA1”^35^ and “TGFB1”^36^. Among the other genes that have not been reported to be associated with CRC metastasis, we selected two genes (OI4, UPA) to explore their role in metastasis. We used the loss-of-function antisense approach. The shRNA lentivirus was used to knock down the expression of these two genes in HCT116 cell line, respectively. Western blotting demonstrated that lower expression of these two genes, respectively, had lower proliferation ability than the control group (Fig. 4a). Furthermore, in vitro cell wound-healing assays revealed that lower expression of these two genes, respectively, could also reduce the migration power in HCT116, compared with controls (Fig. 4b). Overall, these suggest that these two genes regulated the metastatic potential of colon cancer in vitro.

### Genomic characteristics of the different prognostic clusters

For the 227 stage II patients with RNA-seq profiles, 189 and 221 samples also have somatic mutation and CNV data, respectively. These multi-omics datasets allowed us to characterise the genomic features of the two risk clusters.

**Fig. 5:**
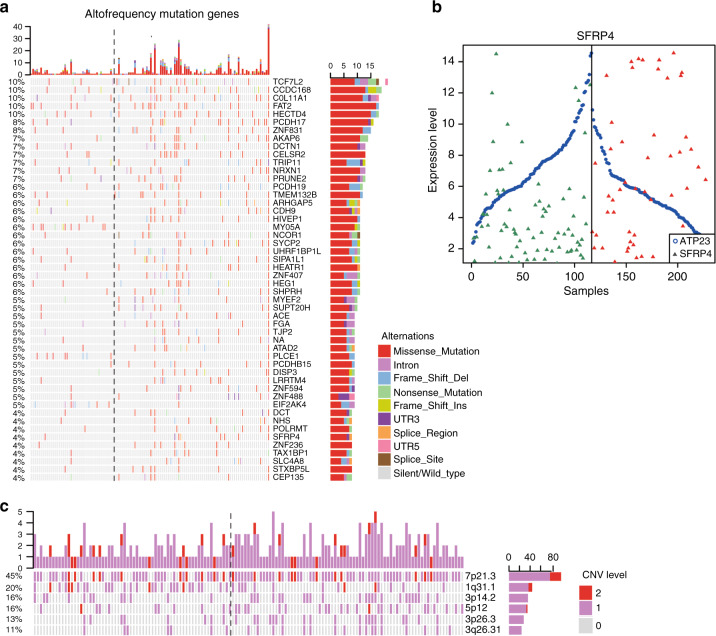
Multidimensional omics analysis between high- and low-risk samples divided by 51-GPS. **a** Differences in the frequency of non-silent mutant genes and **c** significant CNVs between the two risk clusters. Genes with non-silent variants in top 50 and significant genomic regions were depicted on the OncoPrint. Bars on top and to the right of the graph show the number of mutant genes or CNVs in each patient and gene, respectively. The left side of the dotted line represents the low-risk samples predicted by 51-GPS; the right side represents the high-risk samples. **b** Comparison of expression levels of signature gene SFRP4 with paired gene ATP23 between high- and low-risk samples. The blue bots represent reference gene (ATP23), green triangles represent the expression of SFRP4 in low-risk samples and red triangles represent the expression of SFRP4 in high-risk samples.

For the 189 samples with somatic mutation data, 93 and 96 were classified into low- and high-risk samples, respectively. We identified 210 genes that had significantly different mutation frequencies between the two risk clusters (Fisher’s exact test, *p* < 0.05). Especially, 203 of 210 genes had significantly higher mutation rates in the high- than in the low-risk cluster, suggesting that high-risk samples had an increased degree of genomic instability (Fig. 5a). Furthermore, some of the highly frequently mutated genes have been reported to increase the relapse risk of CRC patients. For example, PIK3C2A, missense mutated in 5.2% of high-risk cluster, plays roles in cell proliferation, migration and intracellular protein trafficking.^37^ Another gene, CDH9, encoding a type II classical cadherin from the cadherin superfamily, and mediating calcium-dependent cell–cell adhesion, can contribute to the procession of EMT and lead to a poor outcome.^38^ Notably, two EMT-related genes, TCF7L2 and SFRP4, also showed significantly higher mutation frequencies in the high-risk cluster. TCF7L2 was found to be under-expressed in the high-risk cluster, the loss-of-function mutation of which has been reported to be strongly associated with the risk of CRC.^39^ Conversely, SFRP4 was overexpressed and may be a gain-of-function mutation in the high-risk cluster.^40^ Genes co-expressed with SFRP4 in stage II patients of the TCGA cohort were enriched in “epithelial–mesenchymal transition signalling” and EMT-related functional gene sets (GSEA analysis), such as “apical junction” and “interferon gamma signalling”, suggesting that SFRP4 may be a driver gene for metastasis of CRC patients. Furthermore, as a signature gene, the expression of SFRP4 was reversed compared with reference genes between high- and low-risk samples (Fig. 5b).

For the 221 samples with CNV data, we found six genomic regions, containing three amplification regions and three deletion regions, with significantly different CNV frequencies between the 93 low- and 96 high-risk cluster (Fisher’s exact test, *p* < 0.05, Fig. 5c). Importantly, 54 EMT-related genes are located in these chromosome regions. For example, AGR2 is a member of protein disulfide isomerase (PDI) family, located at chromosome 7p21.3, with significant amplification frequency that promotes migration of CRC cells.^41^ PPARG located at chromosome 3p26.3, with significantly high deletion frequency and low expression level, has been identified as an important step in CRC progression.^42^ RAF1 also located at 3p26.3 is a proto-oncogene that serves as a pivotal member downstream of epidermal growth factors, whose copy number deletion influences cell growth, survival and differentiation.^43^ Besides, the loss of 3p26.3 is an independent prognostic factor in patients with oral squamous cell carcinoma.^44^ Especially, our study demonstrating that SFRP4 had not only high-frequency mutation and significantly high expression, but also significant copy number variation in high-risk samples, reinforces the proposed function of driving metastasis for SFRP4 in stage II CRC.

In conclusion, the high-risk samples predicted by 51-GPS had high-frequency mutation and copy number variants, which will lead to poor outcomes.

## Discussion

EMT has been reported to play a crucial role in mediating tumour metastasis. In this study, based on the hypothesis that the stage II patients who relapse after surgery could be primarily attributed to micrometastasis, we developed a gene pair signature using EMT-related genes for predicting post-surgery relapse risk of stage II CRC patients. The signature showed robust prognostic efficiency across different platforms, and achieved better predictive performance than other known signatures. Although the IRGPI was also constructed by REO-based individualised prognostic method, it did not perform predictive capacity for the RNA-seq dataset from TCGA, and it was validated without considering the specificity of stage. This suggests that EMT may play a more important role in relapse of stage II CRC than immune micro-environmental changes.

The prognostic signature associated with EMT may open up a special perspective for exploring the mechanism of relapse for stage II CRC. The GSEA analysis indicted that EMT-related functional gene sets achieved high enrichment scores in high-risk samples. Multidimensional omics analysis demonstrated that some EMT-related genes had high-frequency mutation and CNVs in high-risk samples, which may be driver genes in micrometastasis of CRC. Furthermore, loss-of-function antisense approach showed that metastasis-related genes between high- and low-risk cluster regulated the metastatic potential of colon cancer in vitro. All the results could illustrate the role of EMT in the relapse, that is, micrometastasis of stage II CRC.

In conclusion, the EMT-related prognostic signature can be a useful predictive tool to figure outpatients at high risk of relapse, and can facilitate personalised management of patients with stage II CRC. Besides, by analysing the molecular changes between two risk clusters, we revealed the potential molecular mechanisms for differences in the risk of relapse in stage II CRC.

## Supplementary information

Supplementary Tables

## Data Availability

All data analysed in this study were downloaded from the public database: Gene Expression Omnibus (GEO, https://www.ncbi.nlm.nih.gov/geo/) and The Cancer Genome Atlas (TCGA, http://cancergenome.nih.gov/).
