# Supplementary material for: Identification of EMT-related high-risk stage II colorectal cancer and characterisation of metastasis-related genes
Source: Br J Cancer. 2020 May 21;123(3):410–7. doi: 10.1038/s41416-020-0902-y (PMC7403418; doi:10.1038/s41416-020-0902-y)
Supplement: Supplementary file 1 — Supplementary Tables [file 41416_2020_902_MOESM1_ESM.doc]

**Supplementary Table S1a: Datasets used in this study.**

| Database | | Platforms | Stage1 | Stage2 | Stage3&4 |
| --- | --- | --- | --- | --- | --- |
| **Discovery datasets** | | | | |  |
| GSE39582 | | HG-U133_Plus_2 | 38 | 208 | 72 |
| TCGA | | Illumina HiSeq | 103 | / | 263 |
| **Validated datasets** | | | | |  |
| TCGA | | Illumina HiSeq |  | 205 |  |
| GSE14333 | | HG-U133_Plus_2 |  | 85 |  |
| GSE17538 | |  |  | 70 |  |
| GSE33113 | |  |  | 89 |  |
| Com_570 | GSE26906 | HG-U133_Plus_2 |  | 21 |  |
|  | GSE31595 |  |  | 14 |  |
|  | GSE39084 |  |  | 6 |  |
|  | GSE92921 |  |  | 43 |  |
| Com_96 | GSE12945 | HG-U133A |  | 4 |  |
|  | GSE41258 |  |  | 22 |  |
| GSE50760 | | Illumina HiSeq 2000 |  |  | 54  (18primary  18metastasis  18normal) |

**Supplementary Table S1b: Specific clinicopathological features of the datasets used in this study.**

|  |  | GSE39582 | GSE14333 | GSE33113 | GSE17538 |
| --- | --- | --- | --- | --- | --- |
| Location |  |  |  |  |  |
|  | Distal | 119 | 41 |  |  |
|  | Proximal | 89 | 43 |  |  |
|  | N/A | 0 | 1 |  |  |
| Gender |  |  |  |  |  |
|  | Male | 123 | 45 | 42 | 34 |
|  | Female | 85 | 40 | 47 | 36 |
| Age (mean) |  | 24-94(70.1) | 30-92(67.4) | 34.6-95.1(70.3) | 38-94(67.2) |
| MMR |  |  |  |  |  |
|  | dMMR | 34 |  |  |  |
|  | pMMR | 144 |  |  |  |
|  | N/A | 30 |  |  |  |
| CIMP |  |  |  |  |  |
|  | + | 37 |  |  |  |
|  | - | 149 |  |  |  |
|  | N/A | 22 |  |  |  |
| CIN |  |  |  |  |  |
|  | + | 126 |  |  |  |
|  | - | 54 |  |  |  |
|  | N/A | 28 |  |  |  |
| TP53 |  |  |  |  |  |
|  | Mutation | 50 |  |  |  |
|  | Wild-type | 59 |  |  |  |
|  | N/A | 99 |  |  |  |
| KRAS |  |  |  |  |  |
|  | Mutation | 67 |  |  |  |
|  | Wild-type | 128 |  |  |  |
|  | N/A | 13 |  |  |  |
| BRAF |  |  |  |  |  |
|  | Mutation | 20 |  |  |  |
|  | Wild-type | 168 |  |  |  |
|  | N/A | 20 |  |  |  |
| Grade | 1 |  |  |  | 5 |
|  | 2 |  |  |  | 52 |
|  | 3 |  |  |  | 6 |
|  | N/A |  |  |  | 7 |

**Supplementary Table S2: The prognostic performance for 3-GPS and 51-GPS.**

| Signature | Datasets | GSE14333 | GSE17538 | GSE33113 | TCGA |
| --- | --- | --- | --- | --- | --- |
| 3-GPS | p-value | 3.82E-04 | 0.193 | 4.05E-04 | 0.504 |
|  | C-index | 0.748 | 0.579 | 0.556 | 0.512 |
|  | HR | 9.15 | 2.22 | 9.67 | 0.79 |
|  | 95%CIs | 2.13-39.2 | 0.65-7.59 | 2.07-45.15 | 0.4-1.57 |
| 51-GPS | p-value | 0.073 | 0.007 | 0.011 | 1.98E-04 |
|  | C-index | 0.639 | 0.71 | 0.656 | 0.639 |
|  | HR | 3.91 | 9.89 | 3.32 | 3.6 |
|  | 95%CIs | 0.78-19.48 | 1.26-77.38 | 1.24-8.88 | 1.76-7.38 |

**Supplementary Table S3a: The prognostic signature 51-GPS.**

| Signature | *Ga* | *Gb* | Signature | *Ga* | *Gb* |
| --- | --- | --- | --- | --- | --- |
| Pair1 | *SERPINE1* | *NUP58* | Pair27 | *CALU* | *PARM1* |
| Pair2 | *SERPINE1* | *TTC27* | Pair28 | *CTHRC1* | *NEDD8* |
| Pair3 | *VEGFA* | *UGT8* | Pair29 | *SNX19* | *MECOM* |
| Pair4 | *FSTL3* | *DALRD3* | Pair30 | *SERPINE1* | *GUF1* |
| Pair5 | *IGFBP3* | *USO1* | Pair31 | *MVD* | *NEO1* |
| Pair6 | *SERPINE1* | *SDAD1* | Pair32 | *IGFBP3* | *TPD52* |
| Pair7 | *SERPINE1* | *AGFG1* | Pair33 | *SERPINE1* | *SFXN1* |
| Pair8 | *SERPINE1* | *CCDC174* | Pair34 | *THBS2* | *SMIM20* |
| Pair9 | *IGFBP3* | *CRK* | Pair35 | *UPP1* | *CALML4* |
| Pair10 | *UPP1* | *USP38* | Pair36 | *SERPINE1* | *RNF213* |
| Pair11 | *COL18A1* | *SLC30A9* | Pair37 | *MCAM* | *MINPP1* |
| Pair12 | *MVD* | *EPB41L4B* | Pair38 | *RARA* | *MECOM* |
| Pair13 | *KIAA1217* | *MECOM* | Pair39 | *AXL* | *TRAF3* |
| Pair14 | *IGFBP3* | *TOP2B* | Pair40 | *ITGA5* | *CNOT6* |
| Pair15 | *SERPINE1* | *DHX8* | Pair41 | *FSTL3* | *FAM241B* |
| Pair16 | *SERPINE1* | *NAAA* | Pair42 | *MPV17L2* | *GIPC2* |
| Pair17 | *SERPINE1* | *ELMO2* | Pair43 | *COL1A1* | *EIF5A* |
| Pair18 | *FSTL3* | *SLC25A17* | Pair44 | *COMP* | *LYSMD1* |
| Pair19 | *MAP4K4* | *TCF12* | Pair45 | *THY1* | *HDHD5* |
| Pair20 | *SERPINE1* | *PKP4* | Pair46 | *LOXL2* | *MECOM* |
| Pair21 | *SERPINE1* | *CCHCR1* | Pair47 | *SFRP4* | *ATP23* |
| Pair22 | *SERPINE1* | *WIZ* | Pair48 | *FSTL3* | *DCAF10* |
| Pair23 | *FSTL3* | *SUGP1* | Pair49 | *VAT1* | *CSK* |
| Pair24 | *ITGA5* | *APOL2* | Pair50 | *MAPK1* | *GLCE* |
| Pair25 | *SERPINE1* | *SLC25A4* | Pair51 | *TAGLN* | *JAGN1* |
| Pair26 | *IGFBP3* | *C9orf152* |  |  |  |
| Note: A sample was classified as high-risk cluster if at least 25 gene pairs voted for high-risk, otherwise, low-risk cluster. | | | | | |

**Supplementary Table S3b: The prognostic signature 39-GPS.**

| Signature | *Ga* | *Gb* | Signature | *Ga* | *Gb* |
| --- | --- | --- | --- | --- | --- |
| Pair1 | *SERPINE1* | *NUP58* | Pair21 | *ITGA5* | *APOL2* |
| Pair2 | *SERPINE1* | *TTC27* | Pair22 | *SERPINE1* | *SLC25A4* |
| Pair3 | *VEGFA* | *UGT8* | Pair23 | *CALU* | *PARM1* |
| Pair4 | *FSTL3* | *DALRD3* | Pair24 | *SNX19* | *MECOM* |
| Pair5 | *IGFBP3* | *USO1* | Pair25 | *SERPINE1* | *GUF1* |
| Pair6 | *SERPINE1* | *SDAD1* | Pair26 | *MVD* | *NEO1* |
| Pair7 | *SERPINE1* | *AGFG1* | Pair27 | *IGFBP3* | *TPD52* |
| Pair8 | *IGFBP3* | *CRK* | Pair28 | *SERPINE1* | *SFXN1* |
| Pair9 | *COL18A1* | *SLC30A9* | Pair29 | *UPP1* | *CALML4* |
| Pair10 | *MVD* | *EPB41L4B* | Pair30 | *MCAM* | *MINPP1* |
| Pair11 | *IGFBP3* | *TOP2B* | Pair31 | *RARA* | *MECOM* |
| Pair12 | *SERPINE1* | *DHX8* | Pair32 | *AXL* | *TRAF3* |
| Pair13 | *SERPINE1* | *NAAA* | Pair33 | *ITGA5* | *CNOT6* |
| Pair14 | *SERPINE1* | *ELMO2* | Pair34 | *COL1A1* | *EIF5A* |
| Pair15 | *FSTL3* | *SLC25A17* | Pair35 | *THY1* | *HDHD5* |
| Pair16 | *MAP4K4* | *TCF12* | Pair36 | *LOXL2* | *MECOM* |
| Pair17 | *SERPINE1* | *PKP4* | Pair37 | *FSTL3* | *DCAF10* |
| Pair18 | *SERPINE1* | *CCHCR1* | Pair38 | *VAT1* | *CSK* |
| Pair19 | *SERPINE1* | *WIZ* | Pair39 | *MAPK1* | *GLCE* |
| Pair20 | *FSTL3* | *SUGP1* |  |  |  |

Note: A sample was classified as high-risk cluster if at least 20 gene pairs voted for high-risk, otherwise, low-risk cluster.

**Supplementary Table S4. Twenty-two gene sets enriched in high-**risk cluster.

| HALLMARK GENE SET | ES | NES | Nom *p*-value | Adjusted *p*-value |
| --- | --- | --- | --- | --- |
| HALLMARK_EPITHELIAL_MESENCHYMAL_TRANSITION | 0.794 | 2.328 | 0 | 0 |
| HALLMARK_COAGULATION | 0.654 | 2.377 | 0 | 0 |
| HALLMARK_APICAL_JUNCTION | 0.617 | 2.279 | 0 | 0 |
| HALLMARK_KRAS_SIGNALING_UP | 0.579 | 2.237 | 0 | 2.13E-04 |
| HALLMARK_INFLAMMATORY_RESPONSE | 0.675 | 2.225 | 0 | 1.71E-04 |
| HALLMARK_COMPLEMENT | 0.595 | 2.201 | 0 | 3.37E-04 |
| HALLMARK_IL2_STAT5_SIGNALING | 0.565 | 2.198 | 0 | 4.61E-04 |
| HALLMARK_MYOGENESIS | 0.637 | 2.166 | 0 | 5.45E-04 |
| HALLMARK_IL6_JAK_STAT3_SIGNALING | 0.675 | 2.163 | 0 | 4.84E-04 |
| HALLMARK_HYPOXIA | 0.542 | 2.160 | 2.07E-03 | 5.76E-04 |
| HALLMARK_TNFA_SIGNALING_VIA_NFKB | 0.638 | 2.092 | 0 | 1.48E-03 |
| HALLMARK_ALLOGRAFT_REJECTION | 0.644 | 2.060 | 0 | 2.21E-03 |
| HALLMARK_ANGIOGENESIS | 0.691 | 2.050 | 2.04E-03 | 2.37E-03 |
| HALLMARK_UV_RESPONSE_DN | 0.596 | 2.025 | 0 | 2.68E-03 |
| HALLMARK_APOPTOSIS | 0.484 | 1.982 | 0 | 3.63E-03 |
| HALLMARK_HEDGEHOG_SIGNALING | 0.656 | 1.938 | 1.93E-03 | 5.38E-03 |
| HALLMARK_INTERFERON_GAMMA_RESPONSE | 0.639 | 1.909 | 5.89E-03 | 7.26E-03 |
| HALLMARK_NOTCH_SIGNALING | 0.550 | 1.887 | 0 | 8.42E-03 |
| HALLMARK_TGF_BETA_SIGNALING | 0.513 | 1.725 | 7.87E-03 | 2.97E-02 |
| HALLMARK_KRAS_SIGNALING_DN | 0.412 | 1.687 | 0 | 3.65E-02 |
| HALLMARK_APICAL_SURFACE | 0.490 | 1.663 | 9.98E-03 | 4.14E-02 |
| HALLMARK_ESTROGEN_RESPONSE_EARLY | 0.353 | 1.523 | 2.83E-02 | 8.56E-02 |
